# Outcomes of Patients With Cocaine Induced Chest Pain in An Inner City Hospital

**DOI:** 10.4021/cr103w

**Published:** 2011-11-20

**Authors:** Moustapha Atoui, Nadia Fida, Suresh Kumar Nayudu, Mariela Glandt, Sridhar Chilimuri

**Affiliations:** a Department of Medicine, Bronx Lebanon Hospital Center, Affiliated with Albert Einstein College of Medicine, Bronx, New York, USA

**Keywords:** Cocaine, Chest Pain, Outcome

## Abstract

**Background:**

Cocaine induced chest pain is a major reason for admission in Safety Net Hospitals in the United States. The majority of patients admitted undergo extensive work-up leading to enormous economic burden. We hypothesize that in individuals with low risk, cocaine does not further increase adverse cardiovascular outcomes.

**Methods:**

We conducted a retrospective chart review of all patients admitted with chest pain to our hospital between 07/01/09 and 06/30/10. We excluded patients with modifiable risk factors for coronary artery disease (CAD). The study population was divided into cocaine and non-cocaine group based on urine drug screen. We analyzed data including demographic, laboratory, cardiac testing, detection of CAD, length of stay and mortality rates.

**Results:**

A total of 426 individuals matched our inclusion and exclusion criteria and were considered to have no known modifiable cardiac risk factors; 54 in cocaine group and 372 in non-cocaine group. Based on physician discretion, 41(76%) in the cocaine group and 239(64%) in the non-cocaine group underwent various modalities of cardiac testing. Cardiac testing was positive in 6(2.5%) patients in non-cocaine group and none in the cocaine group (p=0.597). There was no significant difference between length of stay and in-hospital mortality between the two groups.

**Conclusions:**

In individuals at low risk for CAD, cocaine use resulted in higher rate of cardiac testing. However, there is no difference in prevalence of CAD and in-hospital mortality between the two groups. We conclude that cocaine does not increase adverse outcomes in patients with low risk for CAD.

## Introduction

Cocaine is a significant problem in urban hospitals, accounting for the most encountered emergency department (ED) visits related to substance abuse in the United States [1, 2]. From 1999 to 2002, the number of these visits increased by 47% [3]. Due to its inherent pharmacokinetic properties, cocaine is known to cause several cardiovascular complications, including myocardial infarction, myocarditis, myopathy, arrhythmias, coronary artery aneurysm formation, stroke, and aortic dissection [4]. These complications appear to be independent of dose and route of administration [5]. Most of the risk of myocardial infarction appears to be within the first few hours following cocaine use [6-8].

In current practice, these patients using cocaine are managed as if they had modifiable risk factors for CAD. These patients also have decreased likelihood of compliance with outpatient follow-up [9]. For these two reasons, most of this population undergoes in-patient cardiac testing once admitted.

The management of these patients, however, is based on limited data, making it a challenge for physicians to risk-stratify them. We were interested in studying whether cocaine should be regarded as a traditional risk factor for heart disease in the subgroup of patients who present to the ER with chest pain with no other risk factors other than cocaine use.

## Methods

We conducted a retrospective chart review of consecutive patients admitted with chest pain to Bronx Lebanon Hospital Center (BLHC) between July 1, 2009 and June 30, 2010. Electronic medical records were reviewed for age, gender, race, body mass index (BMI kg/m^2^), past medical history, length of stay in days (LOS) and in-hospital mortality. Laboratory data including serum cardiac markers Creatinine phosphokinase (CPK), Creatinine kinsase-MB (CK-MB), Troponin-T and urine toxicology screen was extracted. Records were also reviewed for cardiac testing including echocardiogram, stress tests (nuclear stress test, stress echocardiography and exercise stress test) and cardiac catheterization.

We excluded patients who were admitted with ST elevation myocardial infarction (STEMI), Non-ST elevation myocardial infarction (NSTEMI), and patients who had traditional risk factors for heart disease, including patients with medical history of coronary artery disease (CAD), cardiomyopathy, hypertension, diabetes mellitus, dyslipidemia and active smoking. We also reviewed cardiac work-up done prior to our study dates to exclude any patient with known CAD and/or chronic systolic congestive heart failure. This was done to exclude patients with atherosclerotic disease because it would make the group not only high risk for further ischemic events, but also because of the reported higher incidence of cocaine-associated cardiovascular events with coexisting coronary artery disease [6].

CAD was defined as luminal obstruction of 50% or more of coronary arteries and their major branches on cardiac catheterization [10]. Cardiomyopathy was defined as LV ejection fraction less than 40% irrespective of etiology. Abnormal echocardiogram was defined as systolic dysfunction with ejection fraction < 40%, Grade II and above diastolic dysfunction, or any wall motion abnormality. Nuclear stress test was defined as abnormal when reported as ischemic defect, scar, or transient ischemic dilatation. Positive stress echocardiogram is defined as abnormal electrocardiogram and wall motion abnormality reported on test result. Exercise stress test was done using Bruce protocol.

This low risk cohort was then divided into two groups based on positive or negative urine toxicology screen for cocaine.

The data was collected as a Performance Improvement Project in the Department of Medicine at BLHC.

### Statistical analysis

The data was analyzed using SAS JMP 8 version [11]. Mean and standard deviation are calculated for demographics and clinical characteristics of the patients using standard methods. The length of stay (LOS) was compared between two groups by two tailed t-Test for unequal variances. In-hospital mortality between the two groups was compared by Chi-square and Fisher’s exact t-Test and plotted as contingency analysis on a mosaic plot. Statistical significance was defined as P < 0.05.

## Results

In the selected review period of one year, a total of 2783 patients were admitted for chest pain. Of these patients, 289 (10.4%) tested positive for cocaine on urine toxicology screen. Of the total admissions, 2357 (85%) were excluded based on the exclusion criteria mentioned above. The remaining cohort of 426 (15%) patients was divided into two groups based on the result of urine drug screen; 54 (13%) were positive for cocaine and 372 (87%) tested negative for cocaine on urine drug screen. Mean age (SD) in cocaine group was 44 (± 10) years and in non-cocaine group 43 (± 12) years (P = 0.47). Both groups also had equal distribution in mean BMI of 23(± 6) kg/m^2^ (P = 0.89) (Table 1). However, cocaine positive group had more males (59%) when compared to (49%) in non-cocaine group (P = 0.19), more African Americans (54%) when compared to (30%) in non-cocaine group, less Hispanics (41%) when compared to (65%) in non-cocaine group (P = 0.003). Also noted was, individuals in cocaine group were more likely to undergo cardiac testing (76%) than the non-cocaine group (64%) (P = 0.12).

**Table 1:** Characteristics of Total Study Group

	Cocaine group	Non-cocaine group	P-value
Number of subjects	54 (13%)	372 (87%)	
Gender			0.19
Female	22 (41%)	191 (51%)	
Male	32 (59%)	181 (49%)	
Race			0.003
Asian	0 (0%)	5 (1%)	
African American	29 (54%)	110 (30%)	
Caucasian	2 (3.5%)	4 (1%)	
Hispanic	22 (41%)	242 (65%)	
Others	1 (1.5%)	11 (3%)	
Age, Mean (SD) years	44 (10)	43 (12)	0.47
BMI (SD) kg/m^2^	28 (6)	28 (6)	0.89
Length of stay, mean (95% CI) days	3 (2.4 - 3.8)	2.4 (2.1 - 2.7)	0.07
Mortality	0	3	1
Cardiac testing	41 (76%)	239 (64%)	0.12

The group that underwent cardiac testing also showed preponderance towards male gender (56%) in cocaine group when compared to (47%) in non-cocaine group (P = 0.31) and African American race (56%) in cocaine group when compared to (30%) in non-cocaine group (P = 0.01). None of the patients in cocaine group had a positive cardiac test result, whereas 6 patients had abnormal cardiac testing in general group (P = 0.6) (Table 2).

**Table 2:** Characteristics of Individuals Who Underwent Cardiac Testing

	Cocaine group	Non-cocaine group	P-value
Number of subjects	41 (76%)	239 (64%)	0.12
Gender			0.31
Female	18 (44%)	126 (53%)	
Male	23 (56%)	113 (47%)	
Race			0.01
Asian	0 (0%)	4 (1.5%)	
African American	23 (56%)	71 (30%)	
Caucasian	1 (2%)	2 (0.5%)	
Hispanic	17 (42%)	155 (65%)	
Others	0 (0%)	7 (3%)	
Age, Mean (SD) years	45 (9)	44 (12)	0.82
BMI (SD) kg/meter square	29 (7)	28 (6.5)	0.6
Length of stay, mean (95% CI) days	3.5 (2.7 - 4.3)	2.4 (2.3 - 3.0)	0.05
Mortality	0	3 (1%)	1
Positive Cardiac testing	0 (0%)	6 (2.5%)	0.6

The mean length of stay (LOS) (95% confidence interval, CI) was higher in cocaine group, 3 days (2.4 - 3.8) when compared to non-cocaine group 2.4 days (2.1 - 2.7) (P = 0.07), though not statistically significant. LOS increased even further in cocaine positive group who underwent cardiac testing, 3.5 days (2.7 - 4.3) when compared to, 2.6 days (2.3 - 3.0) in the non-cocaine group (P = 0.05).

There were no deaths in cocaine positive group whereas 3 patients died during their hospital stay in the control group (P = 1). Out of these, one patient died of metastatic colon carcinoma and sepsis and two patients died of metastatic lung carcinoma. There were no deaths from cardiovascular causes during hospital stay.

## Discussion

In this study we examined patients with no modifiable cardiovascular risk factors who presented to our ER with complaints of chest pain who were found to test positive for cocaine on a urine toxicology screen. Cocaine is traditionally considered as a risk factor for heart disease and frequently leads to further cardiac testing. Our study, however, showed that in low risk patients further testing may unnecessary since no difference was found between the cocaine and non-cocaine groups.

During our study period, ten percent of patients presenting to the ER with complaints of chest pain were found to actively be using cocaine. This statistic is consistent with the data showing that 85% of cocaine users are centered in urban areas [2], particularly inner city areas like the Bronx.

Our study differs from other studies in that we report a higher mean age (44 years) in patients using cocaine presenting with chest pain [12-16]. The younger age of presentation in other studies (mean age less than 40 years) confers them a null or protective factor by Framingham CAD risk at 10 years. The higher baseline risk of our population still did not warrant further cardiac testing.

Studies show low rates of MI (Myocardial Infarction) and mortality overall in cocaine users. Weber et al [14] reported incidence of AMI to be about 6% with cocaine use in the cohort that has traditional risk factors for CAD, but no complication occurred beyond 12 hrs of presentation with 100% survival at discharge. A more recent work by the same authors [12] showed that none of the patients developed cardiovascular complications during the observation period.

Zimmerman et al found that 3 of 48 cocaine-associated chest pain patients (6%) who were admitted to the Coronary Care Units (CCU) sustained an Acute Myocardial Infarction [17]. Gitter et al reported no acute coronary events in their cohort of 101 cocaine-associated chest pain patients admitted to monitored beds [18]. However these studies did not differentiate between low and high risk patients.

A scientific statement from American Heart Association [1] on Management of Cocaine-Associated Chest Pain and Myocardial Infarction recommends optional stress testing for low to moderate risk individuals after an uneventful 9-12 hours observation period. Our study suggests that this additional testing may be unnecessary, since our low risk cocaine cohort had no excess adverse events.

Our study is limited by the lack of long term follow-up. Furthermore, the modalities used for cardiac testing varied based on clinician discretion. Larger prospective studies will need to be done to further ascertain cocaine’s role as a risk factor for CAD in both low and high risk populations.
